# Using influenza surveillance networks to estimate state-specific prevalence of SARS-CoV-2 in the United States

**DOI:** 10.1126/scitranslmed.abc1126

**Published:** 2020-06-22

**Authors:** Justin D. Silverman, Nathaniel Hupert, Alex D. Washburne

**Affiliations:** 1 College of Information Science and Technology, Penn State University, University Park, Pennsylvania 16802, USA.; 2 Department of Medicine, Penn State University, Hershey, Pennsylvania 17033, USA.; 3 Weill Cornell Medicine, Cornell University, New York, New York 10065, USA.; 4 New York-Presbyterian Hospital, New York, New York 10065, USA.; 5 Department of Microbiology and Immunology, Montana State University, Bozeman, Montana 59717, USA.

## Abstract

Detection of SARS-CoV-2 infections to date has relied heavily on RT-PCR testing. However, limited test availability, high false-negative rates, and the existence of asymptomatic or sub-clinical infections have resulted in an under-counting of the true prevalence of SARS-CoV-2. Here, we show how influenza-like illness (ILI) outpatient surveillance data can be used to estimate the prevalence of SARS-CoV-2. We found a surge of non-influenza ILI above the seasonal average in March 2020 and showed that this surge correlated with COVID-19 case counts across states. If 1/3 of patients infected with SARS-CoV-2 in the US sought care, this ILI surge would have corresponded to more than 8.7 million new SARS-CoV-2 infections across the US during the three-week period from March 8 to March 28, 2020. Combining excess ILI counts with the date of onset of community transmission in the US, we also show that the early epidemic in the US was unlikely to have been doubling slower than every 4 days. Together these results suggest a conceptual model for the COVID-19 epidemic in the US characterized by rapid spread across the US with over 80% infected patients remaining undetected. We emphasize the importance of testing these findings with seroprevalence data and discuss the broader potential to use syndromic surveillance for early detection and understanding of emerging infectious diseases.

## INTRODUCTION

The ongoing severe acute respiratory syndrome–coronavirus 2 (SARS-CoV-2) pandemic continues to cause substantial morbidity and mortality around the world (*1*, *2*). Regional preparation for the pandemic requires estimating the growth rate of the epidemic, the timing of the epidemic peak, the demand for hospital resources, and the degree to which current policies may curtail the epidemic, all of which benefit from accurate estimates of the true prevalence of the virus within a population (*3*). Confirmed cases are thought to be underestimates of true prevalence due to some unknown combination of patients not reporting for testing, testing not being conducted, and false-negative test results. Estimating the true prevalence of SARS-CoV-2 would inform the scale of upcoming surges in hospital demand, the proportion of individuals who remain susceptible to contracting the disease, and estimates of key epidemiological parameters such as the epidemic growth rate and the fraction of infections that are sub-clinical.

The current literature suggests that the predominant symptoms associated with COVID-19 are fever, cough, and sore throat; that is, patients often present with an influenza-like illness (ILI) yet test negative for influenza (*4*, *5*). As COVID-19 often presents with similar symptoms to influenza, existing surveillance networks in place for tracking influenza could be used to help track COVID-19. Outpatient ILI surveillance has proven to be a useful tool for assessing the impact of influenza (*6*, *7*). When combined with the number of providers and patients in a given region, ILI surveillance allows estimation of influenza prevalence and severity (*8*–*14*). Studies of outpatient ILI have repeatedly demonstrated that confirmed influenza case rates underestimate disease burden, likely due to preferential testing of more severe cases (*8*, *9*, *13*, *14*). Together these features suggest that ILI surveillance could provide a crucial tool for estimating COVID-19 prevalence within the US.

Here, we quantified the baseline prevalence of non-influenza ILI in the US over the past 10 years and identified a recent surge of non-influenza ILI starting the first week of March, 2020. This surge of excess ILI correlated with known patterns of SARS-CoV-2 spread across states within the US yet was orders of magnitude larger than the number of confirmed COVID-19 cases reported by the end of March.

## RESULTS

### Influenza-like illness surge

**Fig. 1:**
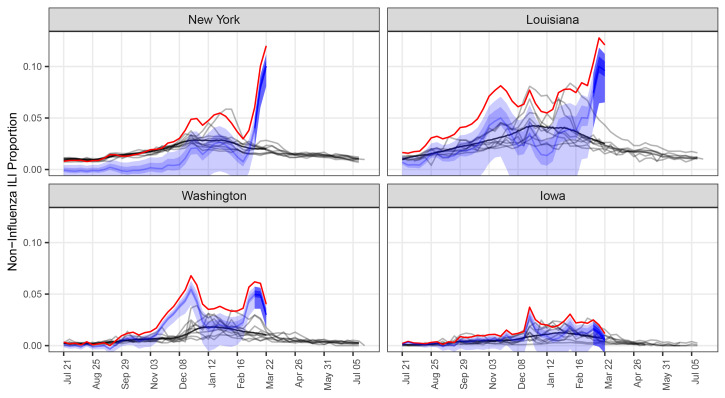
An early surge of ILI visits across the US. The proportion of patients presenting with ILI that could not be explained by influenza or typical seasonal variation (that is, excess ILI) is shown for four states (blue line and ribbons represent the posterior median as well as 95% and 50% credible sets; results from all analyzed states are shown in fig. S1). ILI that could not be attributed to influenza was calculated based on influenza laboratory surveillance data (2019-2020 flu season shown in red, prior seasons are shown in black). A time-series model was used to infer seasonal variation of non-influenza ILI. Excess ILI was then calculated as the difference between non-influenza ILI from 2019-2020 and the seasonal baseline of non-influenza ILI. Excess ILI after March 7th is highlighted in darker blue as these data correlated strongly with observed COVID-19 case counts (fig. S2).

We identified excess ILI cases by first subtracting cases due to influenza and then subtracting the seasonal signal of non-influenza ILI (Fig. 1). Our approach identified known outbreaks of respiratory disease, including the recent outbreak of Respiratory Syncytial Virus that occurred in Washington state in December 2019 (*15*). Starting in March of 2020, many states, including Washington, New York, Oregon, Pennsylvania, Maryland, Colorado, New Jersey, and Louisiana, showed a surge in number of non-influenza ILI cases in excess of seasonal norms. For example, in the fourth week of March, 2020, New York State saw approximately 2 times higher non-influenza ILI than it had ever seen since the inception of the ILINet surveillance system within the US. We found that 10.2% of all outpatient visits in New York State during this time were for ILI that could not be explained by either influenza or the normal seasonal variation of respiratory pathogens (8.0% to 11.2% credible set). As the seasonal surge of endemic non-influenza respiratory pathogens declined toward the later weeks in March, this excess ILI correlated more strongly with state-level patterns of newly confirmed COVID-19 cases, suggesting that this surge is a reflection of ILI due to SARS-CoV-2 (Pearson ρ>0.35 and *p*<0.05 for the last three weeks; fig. S2). The US-wide ILI surge appeared to peak during the week starting on March 15 and subsequently decreased in numerous states the following week; notable exceptions are New York and New Jersey, two of the states that were the hardest hit by the epidemic, which had not started a decline by the week ending March 28.

### Changes in care-seeking behavior

We estimated the ILI surge in each US state as an increase in the proportion of outpatients with ILI in that state compared to all outpatient visits in that state. Consequently, changes in the care-seeking behavior of individuals with ILI or of non-ILI during this time period could each affect our estimates of disease prevalence during the ILI surge. If patients with mild ILI were more likely seek medical care during the month of March 2020 than in prior years, then our estimates of COVID-19 prevalence based on the ILI surge would be falsely elevated. Additionally, if non-ILI patients were less likely to seek medical care during the month of March compared to prior years, then this too could falsely elevate our estimates of COVID-19 prevalence based on the ILI surge. Although ILINet does not provide information to ascertain care seeking behavior, we were able to obtain syndromic surveillance data from New York City’s emergency departments, which provided up to date information on care seeking behavior of both ILI and non-ILI conditions (*16*, *17*).

If the ILI surge reflected higher rates of detection of typically mild ILI, then we would expect emergency department ILI rates to increase yet the proportion of those ILI cases admitted to the hospital to decrease. However, although the daily number of ILI visits to emergency departments across New York City increased in March 2020, the proportion of those patients who went on to be admitted also increased by as much as 3-fold compared to the baseline rate prior to March (fig. S3A). This observation suggests that patients with mild ILI presented less often to hospital emergency departments. Such a decrease in care-seeking behavior for mild ILI, if similar across the US, could deflate the estimated size of the ILI surge in the later weeks of March by a factor of approximately 3.

If non-ILI patients were less likely to seek medical care, then we would expect that the number of patients complaining of other symptoms not typically associated with COVID-19 (for example vomiting) would also decrease compared to prior years. In the month of March, the daily number of patients presenting with vomiting decreased by as much as a factor of 3 compared to the baseline rate in prior years (fig. S3B). Assuming that all non-ILI conditions were similarly decreased during March, this would suggest that our estimates of the ILI surge could be inflated by as much as a factor of 3. This assumption is conservative as it assumes that even individuals with severe conditions (such as severe trauma) would avoid seeking health-care in response to COVID-19 at the same rate as those with more mild conditions such as vomiting. However, the potential 3-fold decreased care-seeking behavior for non-ILI conditions cancels out the potential 3-fold decreased care-seeking behavior of mild ILI, suggesting that our estimates of prevalence based on the ILI surge may be insensitive to recent changes in care-seeking behavior (fig. S3C). Overall these estimates suggest a conceptual model in which health care utilization for both mild ILI and non-ILI conditions declined at similar rates as COVID-19 increased in the US.

### COVID-19 prevalence in the US

To estimate the proportion and magnitude of the March 2020 US ILI surge attributable to SARS-CoV-2 infections, we made the following three assumptions: (1) that the patient population reported by sentinel providers is representative of their state each week; (2) that changes in care-seeking behavior of ILI patients is occurring at a similar rate as that of other non-ILI patients; and (3) that the total number of patients in the US who require medical care over the course of a year has not substantially changed since 2018. Our first assumption is common and underlies prior studies which have used ILI to estimate influenza prevalence (*8*, *14*). Our second assumption is supported by our New York City analysis which suggests that both mild ILI and non-ILI conditions have seen similar changes in healthcare seeking behavior. Our third assumption is based on the observation that the increasing need for health-care between March 8 and March 28, 2020 due to COVID-19 is likely small compared to the approximately 1 billion outpatient encounters that occur annually (*18*, *19*). These assumptions together with surveys describing the average number of patients seen by providers (*19*), the number of providers in each state (*20*), and the total number of outpatient visits per year (*18*, *21*), allowed us to estimate that, if outpatient clinics remained open during the COVID-19 epidemic, we would expect that there would have been approximately 2.8 million patient encounters with ILI due to COVID-19 between March 8 to March 28, 2020 (95% credible set 2.6 million to 3.0 million).

Not all patients infected with SARS-CoV-2 will present to a health-care provider with ILI. Although we cannot directly measure the rate of such sub-clinical cases, a number of prior studies on asymptomatic rates of COVID-19 and the care-seeking behavior of ILI patients in the US suggest a lower-bound on the subclinical rate of patients with ILI. A recent study of passengers on the Diamond Princess cruise-ship accounted for a right-censoring of patients sampled and estimated that 18% of patients infected with SARS-CoV-2 are asymptomatic for the course of their infection (95% credible set 16% to 20%). This estimate likely represents an underestimate given that the majority of passengers were over 60 years old, a demographic thought to have a lower asymptomatic rate than younger individuals (*22*). Beyond asymptomatic individuals, a large study of adult health-care seeking behavior in the United States found that, of a random sample of over 17,000 individuals with ILI, 40% of those went on to seek health care (*23*). Together these additional contributions from sub-clinical cases correspond to a mean clinical rate of 32% (the overall rate at which SARS-CoV-2 cases seek medical care) and a lower bound of 8.7 million SARS-CoV-2 infections between March 8th and March 28th (95% credible set 8.0 million to 9.4 million). Prevalence estimates for each state within this time-period are shown in fig. S4.

### Syndromic case detection rates

We define the syndromic case detection rate as the number of confirmed COVID-19 cases in a week divided by the size of the ILI surge that week. The syndromic case detection rate varied by state and over time (fig. S5). Our estimated syndromic case detection rates increased over the month of March; this was expected given increases in testing capacity across the US since the February 28 detection of community transmission in Washington State. For the week ending March 14, COVID-19 cases in the states with the highest estimated syndromic case detection rate (Washington, Nevada, and Michigan) only captured approximately 1% of ILI surges in those states. In the last week of the month ending on March 28, the syndromic case detection rate across the US increased to 12.5% (95% credible interval 9.5%-18.3%).

### Epidemic growth rates, clinical rates, and infection fatality rates implied by the ILI surge

**Fig. 2:**
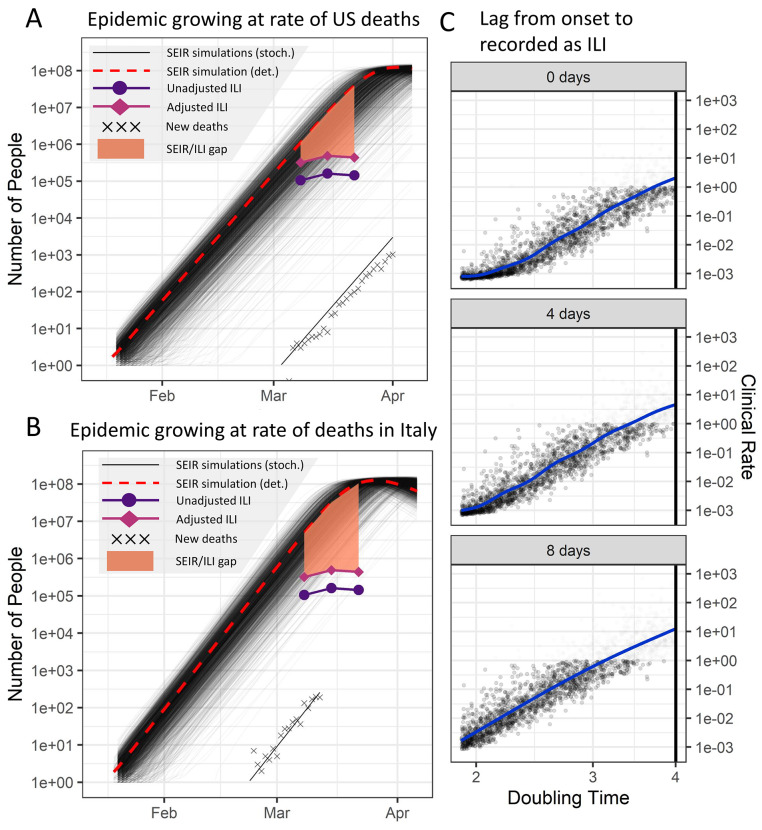
**The ILI surge imposes a dependence between growth rate and clinical rate in epidemiological models** (A-B) SARS-CoV-2 prevalence estimates based on the ILI surge are consistent with an epidemiological model parameterized based on a January 15th epidemic start date and a doubling time equal to that observed for new deaths within the US (A) or Italy (B). Epidemiological models were either stochastic (simulated via tau-leaping) or deterministic (solved by numerical integration). In addition to our raw estimates of the ILI surge size (*unadjusted*), we provide adjusted prevalence estimates accounting for sub-clinical cases by assuming an 18% asymptomatic rate and a 40% rate of health-care seeking of symptomatic ILI patients (*adjusted*). Epidemic trajectories were simulated using an SEIR model (black lines). The increasing gap between ILI prevalence estimates and SEIR trajectories (orange) suggest the presence of additional factors such social distancing, changes in care-seeking behavior, or heterogeneity in susceptibility or transmission. (C) More generally, the size of the clinical population estimated from ILI data imposes a dependence between epidemic doubling time, the clinical rate, and the lag between onset of infectiousness and ILI reporting. Combinations of these three variables that are consistent (black) or inconsistent (gray) are shown as well as a smoothed estimate of clinical rate as a function of doubling time.

The true prevalence of SARS-CoV-2 is unknown at the time of this writing. However, if we assume the excess non-influenza ILI is almost entirely due to SARS-CoV-2, an assumption that becomes more valid as SARS-CoV-2 becomes more prevalent, we can use the excess non-influenza ILI to define lower bounds on the exponential growth rate of the US SARS-CoV-2 epidemic. By estimating the number of patients visiting clinics for COVID-19 in the US in March, we can also identify the mutual dependence of exponential growth rates, the rate of sub-clinical infections, and the time between the onset of infectiousness and a patient reporting as ILI (Fig. 2). Using stochastic Susceptible, Exposed, Infectious and Recovered (SEIR) simulations of US COVID-19 epidemics with a January 15 start date (*24*), we find that an initial epidemic doubling time longer than 4 days is unlikely to explain the ILI surge. Doubling times longer than 4 days fail to produce enough infected individuals to match the observed excess ILI. Doubling time faster than 4 days can explain the observed excess ILI with a clinical rate that depends on the growth rate. Here, we define the clinical rate as the proportion of infected individuals who present to a health care provider.

In keeping with our sub-4 day doubling times, we found that across the entire US, new deaths due to COVID-19 doubled every 3.01 days over the month of March (±0.001, p-value of test that doubling rate is less than 4 days approximately 0). If there was only a 1-day lag from onset of infectiousness to presentation with ILI and the entirety of the first week of the US ILI surge is comprised of patients with COVID-19, then an epidemic starting January 15th and growing at the rate of deaths in the US would imply a 12% clinical rate (Fig. 2A). A four-day lag between the onset of infectiousness and presentation with ILI yields a clinical rate of 25% among the 87% of simulations which could account for the ILI surge. The 25% overall clinical rate estimated from a January 15 start date and the doubling time of US COVID-19 deaths is in close agreement with the 32% clinical rate we estimated independently based on a 18% asymptomatic rate and 40% symptomatic clinical rate. Although our epidemic model suggests the first week of the ILI surge is consistent with the US epidemic start date and growth rate, the ILI surge across the US peaked the week ending March 21, much earlier than our epidemic models, suggesting the epidemic in the US differed from the SEIR model through some combination of factors. Such factors could include successful interventions, even faster decreases in care-seeking than observed in New York, heterogeneity in susceptibility (*25*), or an early epidemic doubling faster than every 3 days.

Faster growth rates require lower clinical rates to explain the ILI surge. Epidemic curves growing at the rate of deaths in Italy, doubling every 2.65 days, could better match the curvature of the ILI surge by peaking around mid to late March, but would imply a clinical rate of 4.7% the second week of March with a 4-day lag between onset and recorded as ILI (Fig. 2B and C). If the entirety of the ILI surge was attributable to COVID-19, the slowest-possible doubling time for the US epidemic which can explain the ILI surge would be a doubling time of of 4 days. Any evidence of significant secondary introductions, super-spreading, or rapid transmission events in early transmission chains will decrease these estimated clinical rates (*26*). Evidence of slow initial spread would increase the estimated clinical rates.

Last, estimating the infection fatality rate from the ILI surge requires knowing the clinical rate and the delay from clinical presentation with ILI to death. If patients present with ILI at the onset of their illness, exhibit a 16 day median lag between onset and death (*27*), and have a 32% clinical rate as estimated from the 18% asymptomatic rate and 40% clinical rate of symptomatic COVID-19 cases, then the observed ILI surge corresponds to an infection fatality rate of 0.29%. We stress that estimating the infection fatality rate from this ILI surge is highly sensitive to both the lag from presentation with ILI to death and the clinical rate (fig. S6). Consequently, the ILI surge is compatible with fatality rates ranging from 0.07% to 1.4% depending on the unknown sub-clinical rate and lag from presentation with ILI to death. Under the CDC planning scenarios specifying a 4-day lag from onset of symptoms to presentation to the doctor with ILI (*28*) and a 15 day lag from onset to death, the resulting 11-day lag from ILI to death produces IFR estimates of 0.57% (0.51-0.68% 95% credible set) for the unadjusted ILI surge and 0.19% (0.17-0.22% 95% credible set) for the ILI surge adjusted to account for asymptomatic and subclinical cases.

## DISCUSSION

We use outpatient ILI surveillance data from around the US to estimate the prevalence of SARS-CoV-2. We found a clear, anomalous surge in ILI outpatients during the COVID-19 epidemic that correlated with the progression of the epidemic in multiple states across the US. The surge of non-influenza ILI outpatients was much larger than the number of confirmed case in each state, providing evidence of large numbers of probable symptomatic COVID-19 cases that remained undetected. This result is also consistent with ILI excess observed in France in late-February/early-March (*29*). Additionally, this finding predicts that the slowest epidemic doubling time that could explain the ILI surge would be 4 days, and that this rate could only be achieved with unusually fast early transmission or super-spreading events and a clinical rate near 100%. Consistent with this prediction, we found that deaths due to COVID-19 within the US doubled every 3.0 days and note that this empirical growth rate for the US epidemic can account for the ILI surge with a 25% clinical rate assuming a 4 day lag from the onset of infectiousness to presentation as an outpatient with ILI. Together, these results suggest that SARS-CoV-2 spread rapidly throughout the US since its January 15th start date and was likely accompanied by a large undiagnosed population of potential COVID-19 outpatients with presumably milder distribution of clinical symptoms than estimated from prior studies of SARS-CoV-2+ inpatients.

Excess ILI appears to have peaked during the week starting on March 15th, leading the observed ILI dynamics to diverge from the overall epidemic dynamics implied by the growth rate of COVID-19 deaths in the US. If the ILI dynamics were proportional to the epidemic curve then the two could be related via a constant subclinical rate. However, the changing ratio between COVID-19 prevalence estimated by the ILI surge and the epidemic curves parameterized by the growth rate of US deaths suggests additional mechanisms may be behind the ILI slowdown. Mechanisms which can explain the difference between our simulated epidemic curves and the ILI surge include effective social distancing, disproportionate reductions in ILI care-seeking behavior relative to non-ILI care-seeking behavior, or heterogeneity in susceptibility or contact structure not captured in our SEIR model (*25*).

Our empirical estimate of the size of the ILI surge has several potential limitations. First, the observed ILI surge may represent more than just SARS-CoV-2 infected patients. A second epidemic of a non-seasonal pathogen that presents with ILI could confound our estimates of ILI due to SARS-CoV-2. However, this seems unlikely as additional viral surveillance through the US Centers for Disease Control and Prevention (CDC) suggests that between March 8 to March 28 other monitored respiratory viruses were at low prevalence (*30*). Nonetheless, were our approach to be used during winter months, additional steps would be needed to account for concomitant non-influenza seasonal pathogens. Additionally, our assumption that changes in health-care seeking behavior are similar between mild ILI and non-ILI condition may be incorrect. Although this assumption was supported by New York City emergency department surveillance data, it is possible that differential health-care seeking would be present in other locations or in the outpatient setting. Last, it is also possible that our use of ILI data has underestimated the prevalence of SARS-CoV-2 within the US. Although early clinical reports focused on cough and fever as the dominant features of COVID-19 (*5*), other reports have documented digestive symptoms as the complaint affecting up to half of patients with laboratory-confirmed COVID-19 (*31*), and alternative presentations, including asymptomatic or unnoticeable infections, could result in underestimation of SARS-CoV-2 prevalence.

Additionally, our models have several limitations. First, we assumed that ILI prevalence within states can be scaled to case counts at the state level. This is based on the assumption that the average number of cases seen by sentinel providers in a given week is representative of the average number of patients seen by all providers within that state in a given week. Errors in this assumption would cause proportional errors in our estimated case counts and syndromic case detection rate. Second, our US-wide SEIR models vary by growth rate alone and as such may not capture important heterogeneity in susceptibility or transmission as well as regional variation, intervention-induced changes in transmission, or clustering of infection outbreaks. Our models were used to illustrate that the ILI surge is consistent with an estimated growth rate and start date for the US epidemic and to specify the mutual dependency of growth rate, the lag between the onset of infection and presentation to a doctor, and clinical rates. Finer models with regional demographic and case-severity compartments are needed to translate our range of estimated prevalence, growth rate, and clinical rates into actionable models for public health managers. Last, our method of calculating the infection fatality rate relied on assumptions about the clinical rate and the delay from patients recorded as ILI to death. Our clinical rate required using patterns of care-seeking for typical seasonal causes of ILI as did our delay from ILI to death; consequently, neither should be relied on as a definitive source for COVDI-19 and estimating the clinical rate and delay from ILI to death for COVID-19 specifically will reduce the large uncertainty around our ILI-estimated infection fatality rates.

Despite these potential limitations, the ILI surge identified in syndromic surveillance time-series allowed early estimates of COVID-19 prevalence, estimates that were not possible from confirmed case data due to early logistical delays in SARS-CoV-2 testing in the US. Our prevalence estimates are supported by a serosurvey conducted in New York State. We estimated that over 8.3% of New York State residents were infected by SARS-CoV-2 by March 28; on April 23, 2020, New York State announced that 14% of residents had evidence of past infection by SARS-CoV-2 by March 29 at which time the cumulative PCR-confirmed case counts totaled only 0.3% of New York’s population (*32*).

Although an ILI surge tightly correlated with COVID-19 case counts across the US and consistent with the New York State serology strongly suggests that SARS-CoV-2 has potentially infected millions in the US, further laboratory confirmation of our hypotheses are still needed to guide public health decisions. Our findings make testable predictions that one would find relatively high seroprevalence in other states that have already seen an ILI surge and that seroprevalence of individuals infected in March across states is proportional to relative sizes of the states’ ILI surges. A study of ILI patients from mid-March who were never diagnosed with COVID-19 could produce a focused test of our predictions about the number and regional prevalence of undetected COVID-19 cases presenting with ILI during that time. If seroprevalence estimates beyond New York State continue to corroborate our prevalence estimates from syndromic surveillance, this would strongly suggest lower case severity rates for COVID-19 than were assumed in late March by comparing PCR-confirmed case counts to deaths. Further corroboration of our estimates of the magnitude of the ILI surge would suggest ILI and other public time-series of outpatient illness allow early and reliable estimates of crucial epidemiological parameters for rapidly unfolding, novel pandemic diseases. As not all novel pandemic diseases are expected to present with influenza-like symptoms, surveillance of other illnesses that commonly present in the outpatient setting could provide a vital tool for rapidly understanding and responding to novel infectious diseases.

## MATERIALS AND METHODS

### Study design

The goal of our study was to use publicly available data to estimate the number of patients seeking care for non-influenza ILI in excess of seasonal trends during the three weeks spanning March 8 to March 28, 2020 and then use this ILI surge to estimate COVID-19 incidence in March and parameterize epidemiological model growth rates and clinical rates.

The ILI surge detection above produced an excess proportion of patients visiting outpatient providers for non-influenza ILI in each week and each state. To scale up the proportion of patients to a national number of COVID-19 cases, we estimated the number of patients per sentinel provider in the CDC dataset, normalized that number of patients per provider to a number of patients per doctor, and scaled that up by an estimated number of practicing doctors in the US. The result was an estimated number of COVID-19 patients visiting doctors in each state for each week – we called this our “unadjusted” ILI surge.

The unadjusted ILI surge is an under-estimate of COVID-19 prevalence due to only clinical infections, those that seek medical care. We accounted for both asymptomatic infections and symptomatic but sub-clinical infections to produce an “adjusted” ILI surge as our final estimate of COVID-19 incidence in each state and each week. We then used the unadjusted and adjusted ILI surges to estimate syndromic case detection and fatality rates. We also used the unadjusted ILI surge as an empirical observation to evaluate epidemiological modelling of COVID-19 growth rates and clinical rates in the US.

Throughout our methods, we use *i* to denote the index state *i* and let *t* index week *t* (with *t*=0 referring to October 3, 2010; the start of state-specific ILINet surveillance).

### Data sources

Since 2010 the CDC has maintained ILINet for weekly influenza surveillance. Each week approximately 2,600 enrolled providers distributed throughout all 50 states as well as Puerto Rico, the District of Columbia, and the US Virgin Islands, report the total number of patient encounters $n_{it}$ and the total number of which met criteria for influenza-like illness (ILI – dined as a temperature 100F [37.8C] or greater, and a cough or sore-throat without a known cause other than influenza; $y_{it}$.) (*37*). For scale, in the 2018-2019 season ILINet reported approximately 60 million outpatient visits. Coupled to these data are weekly state-level reports from clinical and public health labs detailing the number of patient samples tested for influenza $n_{it}^{\text{flu}}$ as well as the number of these samples which are positive for influenza $y_{it}^{\text{flu}}$. Therefore ILINet data can be thought of as a weekly state-level time-series representing the superimposed prevalence of various viruses which can cause ILI. ILINet data was obtained through the CDC FluView Interactive portal (*33*, *38*).

In addition to ILINet data, we downloaded US State population data for the 2020 year was downloaded from https://worldpopulationreview.com/states/. The number of primary care providers in each state per 100,000 residents $b_{i}$ was obtained from the United Health Foundation (*20*) (*24*). COVID-19 confirmed case counts were obtained from The New York Times’ database maintained at https://github.com/nytimes/covid-19-data. This dataset contains the daily cumulative confirmed case count for COVID-19 for each state $z_{il}$ for day *l*. The dataset of deaths in Italy was downloaded from https://github.com/pcm-dpc/COVID-19 on April 6, 2020.

### Data processing

Within the ILINet dataset, New York City and New York were summed into a combined New York variable representing both New York City and the surrounding state. Due to incomplete data in one or more of the data-sources described above the Virgin Islands, Puerto Rico, The Commonwealth of the Northern Mariana Islands, and Florida were excluded from subsequent analysis. In addition, to match the weekly reporting of ILI from ILINet, daily cumulative confirmed COVID-19 cases were converted to weekly counts of new cases by

$${\tilde{z}}_{it}=\underset{l\in t}{\sum }z_{il}-z_{i\left(l-1\right)}.$$

### Extracting non-influenza ILI signal

To subtract influenza signal from $y_{it}$ we assumed that the population of patients with ILI within a state are the same population that are potentially tested for influenza. This assumption allows us to calculate the number of non-influenza ILI cases as

$${\tilde{y}}_{it}=\left(1-\frac{y_{it}^{\text{flu}}}{n_{it}^{\text{flu}}}\right)y_{it}$$

Mean imputation based on neighboring states was used to address missing values in laboratory influenza quantification. To assess the impact of this model for extracting non-influenza ILI signal, we calculated COVID-19 prevalence without first removing signal from influenza, we found little change in our prevalence estimates (fig. S7). This likely reflects that influenza also demonstrates strong seasonal patterns that can be addressed as discussed below.

### Identifying ILI Surges

We identified ILI surges in$\;{\tilde{y}}_{it}$ by training a model on ${\tilde{y}}_{it}$ for all data prior to July 21, 2019. We then used this model to predict the prevalence of non-influenza ILI $\left({\hat{\pi }}_{it}\right)$ for dates after and including July 21, 2019. We calculated the ILI surge as the difference between the observed proportion of non-influenza ILI ${\tilde{y}}_{it}/n_{it}$ and ${\hat{\pi }}_{it}$.

To account for variation in the number of total patients, we modeled $y_{it}$ as binomial distributed. To account for correlation in non-influenza ILI over time, we use a Gaussian Process model which assumes that weeks that are closer together will have more similar levels of non-influenza ILI. The following model reflects these modeling choices:

$${\tilde{y}}_{it}=\text{Binomial}\left(\pi_{it},n_{it}\right)$$

$$\pi_{it}=\frac{\exp\left(\eta_{it}\right)}{1+\exp\left(\eta_{it}\right)}$$

$$\eta_{it}∼N\left(\lambda_{i}\left(t\right),\sigma^{2}\right)$$

$$\lambda_{i}\left(t\right)=∼GP\left(\theta \left(t\right),\sigma^{2}\Gamma \right)$$

$$\sigma^{2}∼\text{InverseGamma}\left(\upsilon ,\xi \right)$$

θ(t)=θ

$$\Gamma \left(t,\;t+s\right)=\alpha \text{exp}\left(\frac{-s^{2}}{2\rho^{2}}\right)$$

Where GP refers to a Gaussian process. We made the following prior specifications: We set the bandwidth parameter for the squared exponential kernel as ρ=3 representing a strong local correlation in time that died off sharply beyond 3 weeks, α=1 representing a signal to noise ratio of approximately 1, ν=1 and ξ=1 representing weak prior knowledge regarding the overall scale of variation in the latent space. Finally, we set θ=−2.197 representing an off-season prevalence of 0.1% non-influenza ILI. Samples from the posterior predictive density $p\left(\pi_{it}|{\tilde{y}}_{i1},\ldots ,{\tilde{y}}_{iT},n_{i1},\ldots ,n_{iT}\right)$ were collected using the function *basset* from the R package *stray* (*34*); a total of 4000 such samples were collected, for each state, in this analysis. We defined the prevalence of non-influenza ILI in excess of normal seasonal variation as $y_{it}^{*}={\tilde{y}}_{it}/\eta_{it}-{\hat{\pi }}_{it}$.

To investigate whether our results were sensitive to the above model specification, we alternatively used the sample mean and variance from years 2010-2018 as an estimate of typical seasonal non-influenza ILI. Despite not accounting for the binomial count structure of ILI data or correlations in the proportion ILI between weeks, this simpler model resulted in nearly identical prevalence estimates (fig. S8). Still, we used the GP-derived estimates throughout this paper due to their better accounting for the known binomial count and week-to-week correlation structure of ILI-causing pathogen prevalence.

To exclude variation attributable to unseasonably high rates of other ILI causing viruses (such as the outbreak of RSV in Washington state in November-December 2019) we only investigated $y_{it}^{*}$ for weeks after March 7th 2020 as only these later weeks had high correlation to the COVID-19 confirmed case rate (fig. S2).

### Calculating scaling factors to relate ILINet data to COVID-19 cases

As new COVID-19 case counts ${\tilde{z}}_{it}$ represent the number of confirmed cases in an entire state and ILINet data represents the number of cases seen by a select number of enrolled providers, we had to estimate scaling factors $w_{i}$ to enable comparison of ILINet data to confirmed case counts at the state level. Let $\pi_{it}^{*}$ denote the probability that a patient with ILI in state *i* has COVID-19 as estimated from ILINet data. Let $p_{i}$ denote the population of state *i* and let $b_{i}$ denote the number of primary care providers per 100,000 people in state *i*. We translated the inferred proportion of individuals with ILI due to COVID-19 to the state level by considering the average number of patients seen across all providers in the state in a 5-day work-week. In addition, we added a discount factor λ=0.55 to calibrate these estimates with prior reports regarding the total number of outpatient visits per year (*18*). This yielded our estimated number of COVID-19 cases (excess ILI at the state level) as

$$w_{i}=\frac{5b_{i}p_{i}}{10^{5}}m\lambda$$

$$y_{it}^{†}=w_{i}\pi_{it}$$

where *m*=20.2 is the mean number of patients seen by physicians per day (*19*).

### Accounting for sub-clinical infections

To account for the contribution of sub-clinical SARS-CoV-2 infections we used a recent analysis of cohort surveillance from the Diamond Princess (*35*). Monte-Carlo simulations were used to propagate error from our uncertainty regarding potential asymptomatic infections affecting the clinical rate $\delta^{b}$ into our calculation of posteriors for epidemic trajectories. To match posterior estimates, we used quantile matching to parameterize $\delta^{c}∼\text{Beta}\left(\alpha ,\beta \right)$ to achieve a mean of 0.179 and a 95% probability set of (0.155, 0.202). In addition, we took $\delta^{c}=0.4$ based on a large study of adult health-care seeking behavior in the United States (*23*). To account for these sub-clinical contributions we used adjusted scaling factors

$$w_{i}^{*}=\frac{w_{i}}{\left(1-\delta^{c}\right)\left(1-\delta^{b}\right)}$$

### Estimating syndromic case detection rates

Assuming that the majority of SARS-CoV-2 testing within the US has been directed by patient symptoms (*36*), the pool of newly diagnosed SARS-CoV-2+ patients is a subset of the pool of SARS-CoV-2+ patients who are identified as having ILI. Therefore, we calculated the probability that a SARS-CoV-2+ patient with ILI who seeks medical care will be identified as having SARS-CoV-2 as $\delta^{s}=\;{\tilde{z}}_{ij}/y_{it}^{†}$ (fig. S5).

### Estimating infection fatality rates

The exact lag from an outpatient being recorded as ILI to death is unknown, but estimated lag times from onset to death and from hospitalization to death (*27*) can be used to understand the range of implied infection fatality rates from the ILI surge. We calculated the infection fatality rate implied by the ILI surge as a function of the unknown lag from patients being recorded as ILI and death, and we repeat this calculation for both the raw and subclinical rate adjusted ILI estimates. For a lag of *l* days from ILI reporting to death, the infection fatality rate was estimated by dividing the magnitude of the adjusted or raw ILI surge by all new deaths occurring within the dates (2020/03/08 + *l*, ..., 2020/03/28 + *l*). A plot of the fatality rate by lag for raw and unadjusted ILI surges revealed a large range of fatality rates compatible with the ILI surge and highly sensitive to the estimate of lag and clinical rates. One study (*27*) estimated a median 11.2 days from hospitalization to death and 16.1 days from symptom onset to death. For the raw ILI surge estimate, 11 day and 16 day lag times would produce median infection fatality rate estimates of 0.57% and 0.89%, respectively, without adjusting for any subclinical infections; for the subclinical-adjusted ILI surge estimate, these lag times would produce median infection fatality rate estimates of 0.19% and 0.29%, respectively.

### Growth rate estimation

As of April 6, 2020, deaths from SARS-CoV-2 epidemic were still growing nearly exponentially as evidenced by nearly linear growth on a log y axis. Early in the epidemic, estimating exponential growth rates by Poisson regression with a log link function produces accurate estimates of the true growth rate (*37*), and so we estimated growth rates for the US and Italy by Poisson generalized linear models predicting new deaths using date as a quantitative explanatory variable. US COVID-19 deaths from March 5, 2020 to April 1, 2020, were summed by date to calculate national-level statistics. Initially, April 2-5 were included but were found to have anomalously high leverage and were hence excluded from our analysis. We applied the same procedure to COVID-19 deaths in Italy, focusing on deaths from February 24 until March 12. We used the slope from Poisson regression as the estimated exponential growth rate, which yielded a US growth rate of $r_{\text{US}}=0.23$ or a 3.01 day doubling time of the infected population over time and an Italian growth rate of $r_{\text{IT}}=0.26$ or a 2.65-day doubling time of the infected population over time.

### Epidemic simulations and clinical rates

The following SEIR models

$$\dot{S}=\zeta -\beta SI-\omega_{b}S$$

$$\dot{E}=\beta SI-\gamma E-\omega_{b}E$$

$$\dot{I}=\gamma E-\upsilon I-\omega_{i}I$$

$$\dot{R}=\upsilon I-\omega_{b}R$$

were parameterized for the US to a timescale of units days by setting $\zeta =3.23\times 10^{-5}$ corresponding to a crude birth rate of 11.8 per 1000 per year, a baseline mortality rate $\omega_{b}=2.38\times 10^{-7}$ corresponding to 8.685 per 1000 per year, and an infectious mortality rate $\omega_{i}=4.96\times 10^{-4}$ corresponding to a infection fatality rate of 0.5% required to fit US deaths under a 20-day lag from onset to death. Further, we drew a random incubation period γ^–1^ ~LogNormal (1.087,0.153) reflecting empirical estimates of a median 5 days from exposure to symptom onset with 4.2-6 day 95% credible interval (*38*, *39*) which is then offset by 2 days of pre-symptomatic transmission as documented across carefully studied clusters in Singapore (*40*) resulting in a 2.2-4 day 95% credible interval for a log-normally distributed incubation period. Similarly, in each simulation we also drew a random infectious period $\nu^{-1}\sim \text{LogNormal}\left(2.193,0.105\right)$ based on 2 days of pre-symptomatic infectiousness and high viral loads in nasopharyngeal samples (*41*, *42*) combined with persistence of high loads of SARS-CoV-2 that can be cultured up to 7 days after symptom onset (*43*), resulting in our use of a 7.3-11 day 95% credible interval for the infectious period. Finally, we parameterized β to ensure *I*(*t*) grew with a specified exponential growth rate early in the epidemic. We ran a total of 2,000 simulations for each of the two growth rate distributions (US and Italy) analyzed. Growth rates were drawn at random from a normal distribution with standard deviation of 0.1 and centered on $r_{\text{US}}$ and $r_{\text{IT}}$, respectively. To illustrate the mutual dependence between estimates of growth rate, clinical rate, and the lag between the onset of infectiousness to presentation to a doctor with ILI, we ran 2,000 simulations with uniform growth rates in the interval [0.173,0.365] corresponding to a range of doubling times between 1.9 days and 4 days.

Each simulation was initialized with $\left(S,E,I,R,t\right)=\left(3.27\times 10^{8},0,1,0,0\right)$ where time 0 was January 15 and simulations were run until August 5, 2020. The SEIR model was simulated with a Gillespie algorithm through the R package adaptivetau (*44*) on the assumption that a large amount of variation in the epidemic trajectory stems from uncertainty in trajectory of early transmission chains. The number of infected individuals on a given day was the last observed *I*(*t*) for that day, and a weekly pool of infected patients was computed by a moving sum over the number of infected individuals every day for the past week, $I_{w}\left(t\right)=\overset{6}{\underset{k=0}{\sum }}I_{t-k}$.

Defining $Y_{t}=\underset{i}{\sum }y_{it}^{†}$ as the national excess ILI, the clinical rate implied by a given simulation was estimated as

$$\delta^{c}\left(t_{d}\right)=\frac{Y_{t}}{I_{w}\left(t-t_{d}\right)}$$

for a given time delay $t_{d}$ it takes from the onset of infectiousness to a patient reporting to the doctor with ILI.

## Supplementary Materials

stm.sciencemag.org/cgi/content/full/scitranslmed.abc1126/DC1 Figure S1. Excess ILI for each US state. Figure S2. Excess ILI correlates strongly with patterns of newly confirmed COVID-19 cases. Figure S3. Surveillance data from New York City emergency departments. Figure S4: Prevalence of SARS-CoV-2 infections between March 8 and March 28, 2020. Figure S5. Syndromic case detection rates by state. Figure S6: Estimating the infection fatality rate (IFR) of COVID-19 based on the unadjusted ILI surge. Figure S7: Investigating model sensitivity when ILI is modeled without first removing signal from influenza. Figure S8. Investigating model sensitivity when seasonal trends in non-influenza ILI are identified using an alternative statistical model.
